# Three trajectories of implementation of medications for opioid use disorder in primary care

**DOI:** 10.1186/s13722-025-00600-y

**Published:** 2025-09-01

**Authors:** Wayne Kepner, Noel Vest, Emma Risner, Hannah Cheng, Brian Hurley, Hannah Snyder, Mark McGovern

**Affiliations:** 1 Division of Primary Care and Population Health, Department of Medicine, Stanford University School of Medicine, Palo Alto, CA USA; 2 Stanford Center for Dissemination and Implementation, Stanford University School of Medicine, Palo Alto, CA USA; 3 Department of Community Health Sciences, School of Public Health, Boston University, Boston, MA USA; 4 Department of Biostatistics, School of Public Health, Boston University, Boston, MA USA; 5 Department of Family Medicine, University of California, Los Angeles, Los Angeles, CA USA; 6 Department of Medical Social Sciences, Feinberg School of Medicine, Northwestern University, Chicago, IL USA; 7 Department of Family Community Medicine, University of California, San Francisco, San Francisco, CA USA; 8 Department of Psychiatry and Behavioral Sciences, Stanford University School of Medicine, Palo Alto, CA USA

**Keywords:** Buprenorphine, Implementation science, Latent class trajectory analysis, Medication for opioid use disorder, Primary health care

## Abstract

**Background:**

The opioid epidemic has prompted nationwide efforts to expand access to medications for opioid use disorder (MOUD). Primary care settings have been identified as a critical access point for patients who may benefit from MOUD treatments. Despite implementation efforts, there is limited understanding of how MOUD practice capability in primary care settings evolves over time or what factors influence clinic-level implementation trajectories.

**Methods:**

We conducted a longitudinal study of 95 primary care clinics in California from 2019 to 2024. MOUD practice capability was measured using the Integrating Medications for Addiction Treatment in Primary Care (IMAT-PC) index across three timepoints. Using latent class growth analysis, we analyzed implementation growth trajectories and examined their associations with clinic characteristics and MOUD implementation outcomes (e.g., patient reach and provider adoption).

**Results:**

Three distinct implementation trajectory classes emerged: elevated improving (41.0%), moderate improving (47.4%), and low improving (11.6%). All clinics demonstrated improvements in MOUD practice capability over time. Elevated improving clinics primarily consisted of smaller clinics (< 15,000 patients) and achieved significantly higher number of patients receiving MOUD compared to moderate (*p* = 0.03) and low improving clinics (*p* = 0.04). Clinics serving medically underserved populations disproportionately represented the low improving class (*p* < 0.01). Increase in the number of providers prescribing MOUD did not differ significantly across trajectory classes.

**Conclusions:**

Although all clinics increased MOUD capability, we found significant associations between implementation trajectory classes and changes in patients receiving MOUD over time in primary care-based MOUD programs. Implementation supports may be more effective and efficient if selected and delivered based upon clinic contextual factors, particularly in resource-constrained and underserved settings.

## Introduction

The United States opioid epidemic has prompted a massive national effort to enhance treatment options for people with opioid use disorder (OUD) [1–5]. Federal agencies, state governments, and healthcare systems have launched large-scale initiatives to combat this epidemic [6–8]. A central focus of these efforts is expanding access to the three FDA-approved medications for opioid use disorder (MOUD): buprenorphine, methadone, and naltrexone (MOUD) [6, 9]. Despite evidence that MOUD improve patient outcomes and drastically reduce mortality, fewer than 20% of eligible patients receive these medications [10, 11]. Consequently, addressing this substantial treatment gap remains a national priority.

Expanding MOUD delivery in primary care settings has emerged as a critical strategy for increasing access to addiction treatment and reducing the negative effects of OUD. Primary care clinics offer efficient, widespread and integrated infrastructure, and can achieve MOUD retention rates that are comparable to specialty addiction clinics [12]. Patients with OUD are also more likely to present to primary care settings due to lower levels of addiction-related stigma, and the benefit of pre-established patient-provider relationships [13–16]. Buprenorphine prescriptions in primary care more than doubled from 2010 to 2018, which was driven in large part by changes in healthcare regulations and increased prescribing from nurse practitioners and physician assistants [17, 18]. Federally Qualified Health Centers (FQHCs) provide accessible healthcare services to low-income communities, and patients receiving MOUDs in community health settings increased from about 39,000 in 2016 to 181,900 in 2020 [19]. However, significant challenges remain such as provider-based stigma, limited training in addiction or addiction medicine, and workforce shortages [20–22].

Despite the massive investment in expanding MOUD access in primary care settings, little is known about how MOUD practice capability develops over time or what distinct trajectory patterns emerge at the clinic level. Current research has focused primarily on clinical outcomes (e.g., treatment retention or reduced substance use) rather than standard clinic-level implementation outcomes, such as patient reach or provider adoption. Identifying distinct growth trajectories of implementation outcomes are important because they can reveal critical insights into how clinics adopt and sustain new MOUD programs [23]. This gap in research on clinic-level trajectories limits our ability to both identify implementation challenges and develop tailored implementation support or practice change strategies that achieve sustained success of MOUD integration in primary care.

The present study examines how clinic-specific factors (e.g., clinic size, patient demographics) and growth trajectories in MOUD practice capability are associated with provider adoption (number of MOUD prescribers) and patient reach (number of patients receiving MOUD) in safety-net community health center primary care clinics. Specifically, our objectives are to: (1) identify distinct trajectory classes of implementation capability using latent class growth analysis (LCGA) (2), examine how trajectory classes differ by clinic characteristics such as urbanicity, size, and medically underserved designation, and (3) evaluate how these trajectory classes are associated with two implementation outcomes: patient reach and provider adoption. To our knowledge, this is the first study to examine implementation trajectories and their association with MOUD implementation outcomes in primary care settings.

## Methods

### Study design and timeline

We conducted a longitudinal implementation study of 95 primary care clinics participating in the Addiction Treatment Starts Here (ATSH) program [24]. The ATSH program was funded by the California Department of Health Care Services to expand access to medication treatment for people with OUD in California community health centers. Data collection occurred across four cohorts between February 2019 and June 2024: Wave 1 (February 2019-September 2020), Wave 2 (August 2019-September 2020), Wave 3 (March 2021-August 2022), and Wave 4 (February 2023-June 2024). Cohorts were defined by enrollment year and in general received equivalent offerings from cohort to cohort. This MOUD practice change program utilized multiple types of implementation strategies: (1) audit and feedback, which involved routine collection and reporting of performance data back to clinics to inform ongoing quality improvement efforts; (2) a learning collaborative that included structured workshops on organizational change strategies and problem-solving; (3) external facilitation involving on-site coaching visits to support leadership, staff and providers; (4) educational didactic webinars that deepened knowledge; (5) peer forums to enhance peer learning, and (6) structured site visits to foster networking and resource sharing [25].

### Sample selection and study population

A total of 96 primary care clinics that applied to participate in the MOUD practice change program were enrolled following a two-stage selection process. Clinics were screened for eligibility: (1) providing care in California (2), meeting safety-net healthcare organization criteria (3), non-profit/tax-exempt status under 501(C) [3] or governmental/tribal entity (4), provision of comprehensive primary care services, and (5) interest in MOUD implementation and/or expansion. Interest in MOUD expansion was self-reported by clinic per application and ascertained qualitatively through their application. All clinics that applied were interested in MOUD expansion. The sample included both startup clinics that were initiating new MOUD programs with little to no prior prescribing experience, and scale-up clinics that were expanding their existing MOUD services. One clinic withdrew due to prescriber turnover which resulted in a final analytic sample of 95 clinics.

### Data collection and measures

The primary outcome was MOUD practice capability, measured using the validated Integrating Medications for Addiction Treatment in Primary Care (IMAT-PC) index, which assesses seven domains (infrastructure, clinic culture and environment, patient identification and initiating care, care delivery and treatment response monitoring, care coordination, workforce, and staff training and development) related to medication integration in the primary care setting [26, 27]. The IMAT-PC index measures organizational capability for delivering MOUD in primary care settings rather than assessing the clinical quality or patient outcomes of treatment. The IMAT domain scores were averaged with equal weighting to create a total composite score ranging from 1 to 5, with scores of 1 to 2 indicating low capability, 3 indicating moderate capability, and 4 to 5 indicating high capability. The IMAT-PC was utilized across all four waves (2019–2024), with data collected at baseline, midpoint, and endpoint of each wave. This team-based and self-reported assessment tool demonstrated high internal consistency (Cronbach’s α = 0.89). Secondary outcomes included adoption (number of providers actively prescribing MOUD) and patient reach (number of new and established patients prescribed MOUD at each time point) collected quarterly via a secure online portal. This approach aligns with implementation science frameworks that distinguish between implementation outcomes (e.g., reach, adoption) and clinical outcomes (e.g., patient health status, treatment retention) [28–30]. Our primary implementation outcomes were guided by the reach, effectiveness, adoption, implementation and maintenance (RE-AIM) framework [30]. Clinics also reported baseline data on clinic characteristics including geographic location, medically underserved area (MUA) designation, medically underserved population (MUP) designation, organization size (categorized as 0–14,999, 15,000–59,999, or ≥ 60,000 patients), organization type (FQHC, FQHC look-alike, ambulatory care clinic, Health Services clinic, or rural health clinic), and patient insurance type (Medicaid, Medicare, dual eligible, private insurance, and uninsured rates). Medically underserved areas designate geographic regions with shortages of primary care providers, while medically underserved populations identify specific population groups experiencing economic, cultural, or linguistic barriers to healthcare within a defined area.

### Statistical analysis

We used latent class growth analysis to identify distinct patterns of IMAT-PC score trajectories over time using model selection based on Akaike Information Criterion (AIC), Bayesian Information Criterion (BIC), entropy, Lo-Mendell-Ruben (LMR), and clinical meaningfulness [31–33]. LCGA differs from traditional latent class analysis (LCA) in that it classifies individuals based on longitudinal change patterns rather than cross-sectional characteristics, allowing identification of trajectory-based subgroups. Bivariate analyses using chi-square tests for categorical variables and analysis of variance (ANOVA) for continuous variables examined differences between latent classes (hereafter referred to as “trajectory classes”). We conducted post-hoc testing for significant associations identified in bivariate analyses. LCGA computations were completed using MPlus version 8.11, and all other analyses were performed using R version 4.4.3 [34, 35].

## Results

### Clinic characteristics

The study included 95 primary care clinics (Table 1) with 88.4% located in urban or metropolitan areas and 11.6% in rural regions as designated by the Federal Office of Rural Health Policy [36]. Importantly, 30.5% of clinics were designated as medically underserved areas and 6.3% served medically underserved populations [37]. The sample consisted primarily of (68.4%) FQHCs plus FQHC look-alikes (4.2%) followed by ambulatory care clinics (17.9%) and smaller numbers of Indian Health Services clinics (5.3%) and rural health clinics at (4.2%). The organizations varied in size, with 43.2% classified as small (i.e., serving under 15,000 patients), 27.4% as medium (i.e., serving 15,000–59,999 patients), and 29.5% as large (i.e., serving over 60,000 patients). For patient insurance type, we report the median percentage of patients within each clinic. The median percentage of patients on Medicaid across clinics was 65.0% (IQR: 50.0–71.0), followed by uninsured patients at a median of 14.0% (IQR: 8.0–20.0), then Medicare at 9.0% (IQR: 3.0–15.0), with private insurance and dual eligibility each at a median of 3.0% (IQR: 1.0–10.0 and 1.0–7.0, respectively).

**Table 1:** Descriptive statistics for clinic characteristics and patient demographics at baseline (*N* = 95)

Clinic characteristic	*N* (%) / Median (IQR)
Population density	
Urban / metropolitan	84 (88.4%)
Rural (FORHP-designated)	11 (11.6%)
Medically underserved area designation	
No medically underserved area designation	66 (69.5%)
Medically underserved area designation (MUA)	29 (30.5%)
Medically underserved population status	
No medically underserved population designation	89 (93.7%)
Medically underserved population designation (MUP)	6 (6.3%)
Primary care clinic designation	
Federally Qualified Health Center (FQHC)	65 (68.4%)
FQHC look-alike	4 (4.2%)
Ambulatory care clinic	17 (17.9%)
Indian Health Service clinic	5 (5.3%)
Rural health clinic	4 (4.2%)
Organization size	
Small size (0–14,999 patients)	41 (43.2%)
Medium size (15,000–59,999 patients)	26 (27.4%)
Large size (60,000 or more patients)	28 (29.5%)
Organization number of unique patients, Median (IQR)	21,447 (8957–61836)
Patient insurance type, Median % (IQR)	
Patients on Medicaid (%)	65.0 (50.0–71.0)
Patients on Medicare (%)	9.0 (3.0–15.0)
Patients with dual eligibility (%)	3.0 (1.0–7.0)
Patients on private insurance (%)	3.0 (1.0–10.0)
Uninsured patients (%)	14.0 (8.0–20.0)

Note. Percentages may not sum to 100% due to rounding. FORHP - Federal Office of Rural Health Policy. MUA – Medically underserved area; MUP – Medically underserved population; IQR – Interquartile range

**Table 2:** Latent class fit indices and most likely class membership sizes

Model	AIC	BIC	Entropy	Class Sizes (%)	LMR *p*-value
1-class	565.78	578.55	--	100%	--
2-class	465.87	486.30	0.83	48.5%, 51.5%	< 0.01
3-class	425.30	453.40	0.89	41.0%, 47.4%, 11.6%	0.01
4-class	420.57	456.32	0.91	41.1%, 2.0%, 10.5%, 46.3%	0.48

Note: aic = akaike information criterion; bic = bayesian information criterion; LMR = Lo-Mendell-Rubin likelihood ratio test. Lower AIC and BIC values indicate better model fit. Entropy values closer to 1 reflect clearer class separation (values ≥ 0.80 are considered acceptable). A significant Lo-Mendell-Rubin (LMR) p-value (*p* < 0.05) supports improved fit over a model with one fewer class

At baseline, clinics demonstrated variable levels of implementation capability across the IMAT-PC domains. Of the domains, the infrastructure domain showed the highest average score (M = 3.9, SD = 0.8); staff training and development had the lowest (M = 2.4, SD = 1.0). The remaining domains (i.e., clinic culture, patient identification, care delivery, care coordination, and workforce) all showed similar average scores ranging from 3.0 to 3.2. The total IMAT-PC score averaged 3.2 (SD = 0.8), indicating moderate MOUD practice capability across clinics. Regarding implementation outcomes, clinics had a mean of 3.1 (SD = 4.0) actively prescribing providers and served an average of 38 patients with MOUD per clinic during the baseline quarter (SD = 82); the wide range of values (0–18 providers and 0-510 patients) suggest substantial variation in implementation outcomes across clinics.

### Model fit and trajectory classes

We evaluated model fit for the LCGA using multiple established indices, including the AIC, BIC, entropy, and the LMR-LRT (Table 2). These fit statistics supported our selected model, balancing statistical fit and interpretability. In particular, the LMR-LRT provided evidence that the selected number of classes significantly improved model fit compared to models with one fewer class, suggesting meaningful separation of trajectory patterns.

Importantly, LCGA assumes no within-class variance in growth parameters (i.e., fixed slopes and intercepts within each class), meaning within-class normality and homogeneity of variance are not required in the same way as in parametric regression models. Instead, variance is captured between classes. We inspected observed vs. estimated trajectories within each class to assess potential misfit. Given the parsimony and interpretability of the LCGA solution, and lack of theoretical justification for estimating within-class variance, more complex approaches such as growth mixture modeling were not pursued.

We computed one to six LCGA models, starting with a single class and increasing by one up to the final six class model. Five and six-class solutions were discarded due to empty classes and poor model fit respectively. Working back from the four-class model, though the entropy value was acceptable in the four-class model, the LMR p-value indicates that adding the additional class did not improve model fit when compared to the model with one less (*k*-1) class. Thus, examining the three-class model next, we found that the BIC was superior (lowest value), and the entropy and LMR values were acceptable. We also noted that the added class from the two-class model was a small but clinically meaningful class that was low in initial IMAT, but showed the most improvement across time. Thus, considering model fit indices, class sizes, and clinical relevance, the three-class solution was deemed the most parsimonious and clinically relevant.

**Fig. 1:**
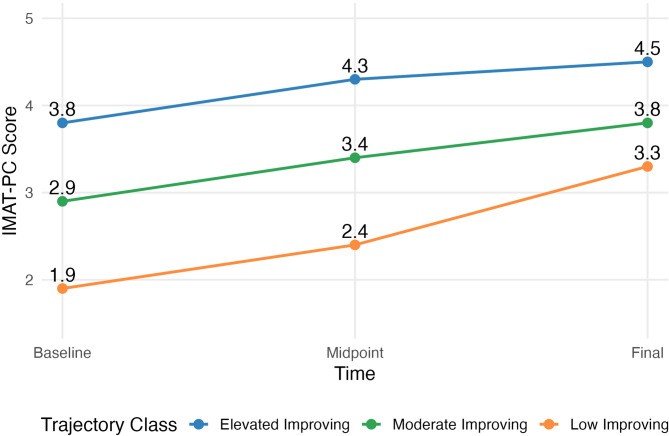
Average IMAT-PC score by timepoint and by trajectory class

Analysis of the 95 primary care clinics revealed three distinct implementation trajectory classes (Table 3; Fig. 1):

**Table 3:** Clinic characteristics by MOUD implementation trajectory: elevated, moderate, and low improving classes (*N* = 95)

Clinic characteristics	Elevated improving *N* (%)	Moderate improving *N* (%)	Low improving *N* (%)	Total *N* (%)	*p*-value
Total number of clinics	39 (41.0%)	45 (47.4%)	11 (11.6%)	95 (100.0%)	
Number of rural clinics	6 (15.4%)	5 (11.1%)	0 (0.0%)	11 (11.6%)	0.37
Medically underserved area (MUA)	11 (28.2%) ᵃᵇ	18 (40.0%) ᵃ	0 (0.0%) ᵇ	29 (30.5%)	0.03*
Medically underserved population (MUP)	3 (7.7%) ᵃᵇ	0 (0.0%) ᵃ	3 (27.3%)ᵇ	6 (6.3%)	< 0.01**
Organization size					0.30
0–14,999 patients	20 (51.3%)	19 (42.2%)	2 (18.2%)	41 (43.2%)	
15,000–59,999 patients	11 (28.2%)	11 (24.4%)	4 (36.4%)	26 (27.4%)	
60,000 + patients	8 (20.5%)	15 (33.3%)	5 (45.5%)	28 (29.5%)	
Organization type					0.16
FQHC or FQHC look-alike	33 (84.6%)	30.0 (66.7%)	6 (54.5%)	69 (72.6%)	
Other	6 (15.4%)	15 (33.3%)	5 (45.5%)	26 (27.4%)	
Site Medicaid proportion (%)^1,2^	64.3 (20.0)	56.4 (15.8)	67.2 (9.9)	61.1 (17.6)	0.07
Site Medicare proportion (%)^1,2^	10.7 (16.4)	11.9 (10.0)	9.9 (6.3)	11.1 (12.8)	0.89
Site dual eligible proportion (%)^1,2^	6.3 (10.2)	4.2 (5.0)	3.9 (4.6)	5.2 (7.9)	0.26
Site private insurance proportion (%)^1,2^	5.6 (7.3)	8.9 (11.8)	3.4 (4.1)	6.8 (9.5)	0.17
Site uninsured proportion (%)^1,2^	13.2 (9.8)	17.0 (12.1)	16.4 (14.3)	15.3 (11.4)	0.32
Reach at baseline: number of patients receiving MOUD^1,2^	51.0 (76.0)	34.0 (93.0)	4.0 (6.0)	38.0 (82.0)	0.25
Reach at endpoint: number of patients receiving MOUD^1,2^	84.0 (105.0) ^a^	39.0 (67.0) ᵇ	13.0 (17.0) ᵇ	54.0 (85.0)	0.01*
Adoption at baseline: number of providers prescribing MOUD^1,2^	3.4 (3.8)	3.4 (4.6)	0.9 (1.0)	3.1 (4.0)	0.18
Adoption at endpoint: number of providers prescribing MOUD^1,2^	5.3 (4.5)	5.6 (9.0)	2.7 (1.8)	5.1 (6.9)	0.57

Note. Chi-Square test unless otherwise specified; ^1^ Indicates ANOVA; ^2^ Indicates Mean (SD); * significant at < 0.05. ** significant at < 0.01. Values within a row not sharing the same superscript letter (a, b) differ significantly based on Tukey HSD post hoc analyses (*p* < 0.05)

Assumptions of normality (Shapiro-Wilks) and homogeneity of variance (Bartlett’s test) were not met for several variables, likely due to small class sizes

Class 1- *Elevated improving clinics* (*n* = 39; 41.0%) demonstrated baseline IMAT-PC scores of 3.8 (SD = 0.3) and improved to 4.5 (SD = 0.3) at program end; this class achieved the highest patient reach (mean = 84.0 patients; SD = 105.0). This class of elevated improving clinics consistently maintained the highest MOUD practice capability and demonstrated steady improvement across measurement periods.

Class 2 - *Moderate improving clinics* (*n* = 45; 47.4%) began with baseline scores of 2.9 (SD = 0.4) and improved to 3.8 (SD = 0.4) at program end which resulted in reaching intermediate patient volumes (mean = 39.0 patients; SD = 67.0). This class showed moderate but consistent improvement while maintaining stable mid-level MOUD practice capability.

Class 3 - *Low improving clinics* (*n* = 11; 11.6%) started with baseline scores of 1.9 (SD = 0.7) and showed the greatest relative improvement, reaching 3.3 (SD = 0.7) at program end but maintained limited patient reach (mean = 13.0 patients; SD = 17.0). Despite having the lowest absolute scores, the low improving class demonstrated the greatest relative improvement in MOUD practice capability and showed a consistent upward trajectory by the program end which suggests that additional gains may emerge beyond our study window.

### Baseline characteristics of implementation trajectory classes

Implementation trajectory class membership was significantly associated with clinic-level characteristics (Table 3). The distribution of clinics serving medically underserved areas varied significantly across trajectory class (*p* = 0.03); moderate improving clinics had the highest proportion (40.0%), followed by elevated improving (28.2%), with no representation (0.0%) in the low improving clinics. Conversely, clinics serving medically underserved populations were disproportionately represented in the low improving class (27.3%) compared with the moderate improving class (0.0%) (p = < 0.01). The elevated improving class included 28.2% MUA and 7.7% MUP designated clinics. In subsequent post-hoc analysis, we found that in the moderate improving class, the proportion of clinics with a designation as serving medically underserved areas was 40.0% (*n* = 18) whereas the low improving class had 0.0% (*n* = 0) of clinics serving medically underserved areas (*p* = 0.03). Conversely, the moderate improving class had 0% (*n* = 0) of clinics with a designation as serving medically underserved populations, whereas over a quarter (27.3%; *n* = 3) of the low improving class serviced medically underserved populations (p = < 0.01).

Organizational size, insurance status, and urbanicity demonstrated interesting patterns but did not reach statistical significance. Small clinics (< 15,000 patients) comprised 51.3% of the elevated improving class, 42.2% of moderate improving, and 18.2% of low improving. Medium-sized clinics (15,000–59,999 patients) represented 28.2% of elevated improving, 24.4% of moderate improving, and 36.4% of low improving classes. Large clinics (≥ 60,000 patients) made up 20.5% of elevated improving, 33.3% of moderate improving, and 45.5% of low improving classes. Medicaid rates varied across trajectory classes (elevated improving: 64.3%; moderate improving: 56.4%; low improving: 67.2%). The proportion of urban clinics (compared with rural clinics) remained consistently high across all trajectory classes (84.6% in elevated improving, 88.9% in moderate improving, and 100.0% in low improving classes).

### Reach and adoption outcomes

We conducted a Tukey Honestly Significant Difference (Tukey HSD) post-hoc analysis to identify significant differences in the three trajectory classes on provider adoption and patient reach outcomes (Table 3). For patient reach, significant differences were observed between the three implementation trajectory classes. Post-hoc analysis revealed that elevated improving clinics achieved significantly higher patient reach compared to moderate improving clinics (mean difference = 45; *p* = 0.03). The difference between elevated improving and low improving clinics showed the largest absolute difference in patient reach (mean difference = 71; *p* = 0.04). The difference between moderate improving and low improving clinics was not statistically significant (mean difference = 40; *p* = 0.84).

For provider adoption, we found no significant differences between the three trajectory classes at either baseline or endpoint. Despite variability in MOUD practice capability across classes, all three classes maintained similar levels of provider adoption throughout the study period. Because the chi-square value for adoption was non-significant, indicating no significant differences on adoption scores at baseline or endpoint, we did not compute post-hoc analysis.

The assumption of normality for continuous variables across latent classes was not met for most classes (as tested by Shapiro-Wilk), likely due to small sample sizes. Homogeneity of variances (Bartlett’s test) was not met for several variables, though Levene’s test resolved most violations except patient reach at the final timepoint, but the variable maintained significant findings in ANOVA testing. We conducted a post hoc power analysis to evaluate the sensitivity of our ANOVA comparisons across trajectory classes, with particular attention to the smallest class (*n* = 11). Using a conservative balanced design approximation, we found that power to detect a medium effect size (Cohen’s *f* = 0.25) was approximately 56%, while power to detect a large effect size (*f* = 0.40) was approximately 94%. Given the unbalanced group sizes in our actual data, these estimates likely overstate power for detecting medium effects, particularly in comparisons involving the smallest class. As a sensitivity analysis, we also conducted non-parametric tests and examined pairwise differences. Results were consistent with the ANOVA findings, supporting the robustness of observed group differences despite the smaller sample size in one class.

## Discussion

Our study identified three distinct implementation trajectory classes among safety-net primary care clinics delivering MOUD: elevated improving, moderate improving, and low improving. These three classes demonstrated significant differences in patient reach outcomes and were significantly associated with specific clinic-level characteristics. Importantly, while all clinics were offered the same implementation support activities and improved MOUD practice capability overall, their growth trajectories varied significantly. Elevated improving clinics achieved significantly higher patient reach compared to other classes, which is underscored by the fact that over half were small-sized clinics (< 15,000 patients). These trajectory patterns align with findings from Hoekstra et al., who identified similar trajectory classes in Dutch rehabilitation clinics [23]. However, unlike Hoekstra et al., who found no association between clinic trajectory classes and implementation outcomes, our study demonstrated significant associations with implementation outcomes (e.g., patient reach). Our findings provide evidence that increasing MOUD practice capability through structured support can substantially increase MOUD access in primary care settings, but clinic-level characteristics may influence implementation outcomes.

The three trajectory classes all showed improvements in MOUD practice capability from baseline. However, clinics differed significantly on patient reach outcomes by the final time point. Elevated improving clinics achieved more than double the number of total patients receiving MOUD compared to moderate improving clinics and more than six times compared to low improving clinics; these results were achieved despite having the highest proportion of small-sized clinics. This demonstrates that clinics with strong baseline MOUD capability and increasing organizational capability can deliver high volumes of MOUD regardless of patient panel size (i.e., the total number of patients). Importantly, patient reach did not significantly differ among the three classes at baseline which supports the possibility that the subsequent differences in reach emerged during the study period. These findings support that MOUD practice change strategies combined with strong organizational capabilities more quickly and directly results in changes in MOUD access for primary care patients in community health settings.

Designation as a medically underserviced clinic was significantly associated with trajectory class. Clinics serving medically underserved areas were overrepresented in the moderate improving class with none in the low improving class; clinics serving medically underserved populations were predominantly in the low improving class. Clinics in MUAs are often rural and may face barriers related to geographic and workforce supply issues, necessitating strategies like telehealth expansion. Conversely, clinics servicing MUPs, which can be urban or rural, face socioeconomic and cultural access issues that require interventions such as culturally competent care and financial assistance. Our findings align with previous research identifying the need for targeted support in resource-constrained settings [38, 39]. However, our findings suggest that even clinics serving medically underserved populations in the low improving class can substantially increase their MOUD practice capability with structured support, and therefore these interventions are valuable to all trajectory classes.

Implementation trajectory class was not associated with significant changes in the number of providers prescribing MOUD (i.e., provider adoption). This contrasts with previous studies showing that structured implementation strategies, including learning collaboratives, significantly enhanced MOUD adoption among providers [40, 41]. Our results suggest that other person-level variables (e.g. provider-based medication stigma), variable clinical expertise, and comfort with addiction treatment may have had a strong influence on adoption [15, 42]. These data show that successful implementation may require targeted approaches that address organizational readiness and provider-level factors through increased training [43] and team-based approaches [44]. Importantly, our study was conducted during the COVID-19 pandemic when telehealth restrictions were loosened and the X-waiver was eliminated which may have affected our adoption-related findings [45, 46].

### Limitations

The observational longitudinal design mitigates causal inferences about the relationships between participation in this practice change initiative, clinic characteristics and implementation outcomes. Data collection during COVID-19 likely influenced our findings through changes in telehealth policy [47]. Potential confounds include variable staff turnover rates and participation in the implementation strategies that were offered. Statistical analysis was limited by the small number of clinics in the low improving class and non-normal distributions of several measures. We used baseline clinic characteristics for the categorization of clinic size, and we did not ascertain changes in patient panel size over time. Program participation varied across clinics and study outcomes (IMAT-PC, patient reach, provider adoption) relied on clinic-level report. Clinics self-selected into the program, potentially biasing toward organizations with greater readiness for change.

## Conclusions

This is the first study to examine implementation trajectory classes in MOUD capability building and their associations with implementation outcomes in primary care settings. Our findings that elevated improving clinics were able to achieve more than double the patient reach of the moderate improving class and more than six times the reach of the low improving class underscores the central role of MOUD practice capability in improving access to MOUDs. However, the disproportionate representation of clinics serving medically underserved populations in the low improving class highlights critical issues related to health equity. These findings have immediate practical implications for expanding MOUD access. Clinics can use the IMAT-PC diagnostically to objectively establish a baseline of current MOUD practice capability and apply it as a continuous measure to track long-term improvement through reassessment. Future research should evaluate how to select and tailor implementation support and practice change strategies to specific clinic characteristics.

## Data Availability

The datasets generated and analyzed during the current study are not publicly available due to confidentiality agreements with participating clinics but are available from the corresponding author on reasonable request.

## Abbreviations

AIC: Akaike Information Criterion
ANOVA: Analysis of Variance
ATSH: Addiction Treatment Starts Here
BIC: Bayesian Information Criterion
FDA: Food and Drug Administration
FQHC: Federally Qualified Health Center
IMAT-PC: Integrating Medications for Addiction Treatment in Primary Care
LCGA: Latent Class Growth Analysis
LMR: Lo-Mendell-Rubin
MOUD: Medications for Opioid Use Disorder
MUA: Medically Underserved Area
MUP: Medically Underserved Population
OUD: Opioid Use Disorder
SAMHSA: Substance Abuse and Mental Health Services Administration
